# Ultra-early (≤8 hours) surgery for thoracolumbar spinal cord injuries: A systematic review and meta-analysis

**DOI:** 10.1016/j.xnsj.2023.100285

**Published:** 2023-10-05

**Authors:** Abhiraj D. Bhimani, Matthew T. Carr, Zahraa Al-sharshai, Zachary Hickman, Konstantinos Margetis

**Affiliations:** Department of Neurosurgery, Icahn School of Medicine at Mount Sinai, One Gustave L. Levy Place, Box 1136, New York, NY 10029, United States

**Keywords:** Meta-analysis, Systematic review, Thoracic spine, Lumbar spine, Thoracolumbar spine, Spinal cord injury, Surgical decompression, Neurological recovery

## Abstract

**Background:**

The impact of the timing of surgery on neurological recovery in thoracolumbar spinal cord injuries (tSCI) is still a subject of discussion. Accumulating evidence is supporting early decompression (<24 hours) following tSCI. However, the potential advantages of earlier decompression remain uncertain. This systematic review and meta-analysis summarize and analyze the current evidence on the effectiveness of ultra-early decompression surgery on clinical outcomes following tSCI.

**Methods:**

A search was conducted in the electronic databases Medline, Embase, Scopus, and Web of Science from their inception until May 2022 for human studies. Groups were stratified into ultra-early (surgery within 8 hours of injury) vs control group operated >8 hours of injury. The authors included the study data from their institutional case series of thoracolumbar spinal cord injury from 2015 to 2018. An arm-based meta-analysis was performed on all studies using the R Studio. For studies that qualified, a contrast-based meta-analysis was also performed with a standardized mean difference (SMD). Outcomes were reported as effect size, treatment effect, and effect difference, all with 95% confidence intervals (CI).

**Results:**

Of the 133 patients, 74.4% patients were male. 76 (57.1%) underwent decompression ≤8 hours, while 57 (42.9%) underwent decompression >8 hours from injury. Quantitative analysis using the SMD model showed a significant difference in mean AIS improvement in the ultra-early group (Effect size 1.15 [0.62–1.67], p<.0001). On arm-based meta-analysis, a statistically significant treatment effect was found for the ultra-early arm (1.25 [0.91–1.67]), while > 8-hour arm did not show significance (0.30 [-0.08-0.71]). There was a statistically significant effect difference between the two arms (0.96 [0.49–1.48]).

**Conclusions:**

This study observed a significant improvement in the mean AIS score in patients undergoing decompression within 8 hours of tSCI. Given the scant literature regarding ultra-early decompression of tSCI, this study solidifies the need to further explore the role of early interventions for tSCIs to improve patient outcomes.

## Introduction

Spinal cord injuries (SCI) remain one of the most devastating neurological disorders, and despite the tremendous interest in these injuries and breakthroughs in our understanding of the pathways implicated in secondary injury cascade, the prognosis for these patients remains dismal [1]. Mortality in SCI can be three times higher than the overall population [2], and survivors are prone to many debilitating complications including respiratory failure, infections, and autonomic dysreflexia.[1] Neurological outcomes after SCI are also poor [3], and there is substantial financial cost associated with SCI [1].

The timing of surgical intervention in SCI and its influence on neurological recovery has been the subject of considerable debate and is one of the most contentious issues in SCI literature. Although evidence from preclinical research indicates that early surgical therapies promote neurological recovery [4], and evidence from retrospective studies is promising in terms of reduced systemic complications and length of hospital stay with early surgery (24 hours), clinical studies remain scant and inconclusive [5]. Some of the existing studies are single-armed and given the limitations surrounding randomization, it is likely that objective comparisons will continue to be unavailable. There is some also data suggesting early decompression is associated with more complications, further clouding the debate [6,7].

In this work, we conduct a comprehensive literature review and run a meta-analysis to estimate the pooled effect of ultra-early surgery, defined as within 8 hours of injury, on neurological recovery in patients with thoracolumbar SCI (tSCI). To the best of our knowledge, this is the first systematic review and meta-analysis on the topic, with the objectives of illustrating the paucity of the literature, highlighting the gaps and deficiencies in existing studies, and ultimately serving as a steppingstone for future research on the topic.

## Methods

### Study design and patient selection

A search was conducted following PRISMA guidelines in the electronic databases Medline, Embase, Scopus, and Web of Science from their inception until May 2022. The authors initiated patient selection by identifying the studies that evaluated surgical intervention after thoracolumbar spinal cord injury. The search included clinical trials and cohort studies. Surgery was considered ultra-early if performed within the first 8 hours of injury. The comparison group was defined as the patients operated after 8 hours of injury. The following exclusion criteria were applied: (1) studies that did not include a separate group for those operated on with 8 hours, (2) improvement not measured by the American Spinal Injury Association Impairment Scale (AIS), (3) letters to editors, commentaries, observational studies, and review articles, (4) nonhuman studies, and (5) articles not published in English. Table 1

**Table 1:** Summary of included studies.

Study	Type	Number of patients	Summary
Cengiz et al.[16]	Prospective randomized study	27	• Patients randomized based on the day of presentation • Ultra-early group: shorter overall hospital and intensive care unit stay, lesser systemic complications such as pneumonia, better neurological improvement
Payer et al.[17]	Prospective observational study	14	• Thoracolumbar burst fractures, 14 of whom had neurological deficits and underwent decompression within 8 h • No control arm
Ramirez et al.[18]	Retrospective study	28	• Patients with thoracolumbar injury with neurological symptoms • 11 patients operated within 8 h, 17 operated after 8 h • Statistically significant mean AIS improvement in patients operated within 8 h
Wutte et al.[19]	Retrospective study	58	• 12-y data from level 1 trauma center • 35 patients operated within 8 h, 23 operated on after 8 h • Early group showed improved AIS grade and higher AIS conversion
Carr at al.	Retrospective case series	6	• Author's institutional case series • 4 patients operated on within 8 h, 2 after 8 h

The publications were assessed by two independent reviewers, and study parameters were collected using a predefined data extraction form. The authors included the study data from their institutional case series of thoracolumbar spinal cord injury from 2015 to 2018. An Institutional review board approval was obtained to use the patient data from the author's institution. The institutional data was referred to as Carr et al. The study protocol was registered at OSF Registries and is freely available to download along with the search terms used in this study at the registration osf.io/n8 × 7w. All the included studies in this meta-analysis are summarized in Table 2.

**Table 2:** GRADE evidence profile for this systematic review and meta-analysis.

Study Limitations	Directness	Consistency	Precision	Reporting bias
High	Direct	Consistent	Precise	Undetected

### Data collection and analysis

**Fig. 1:**
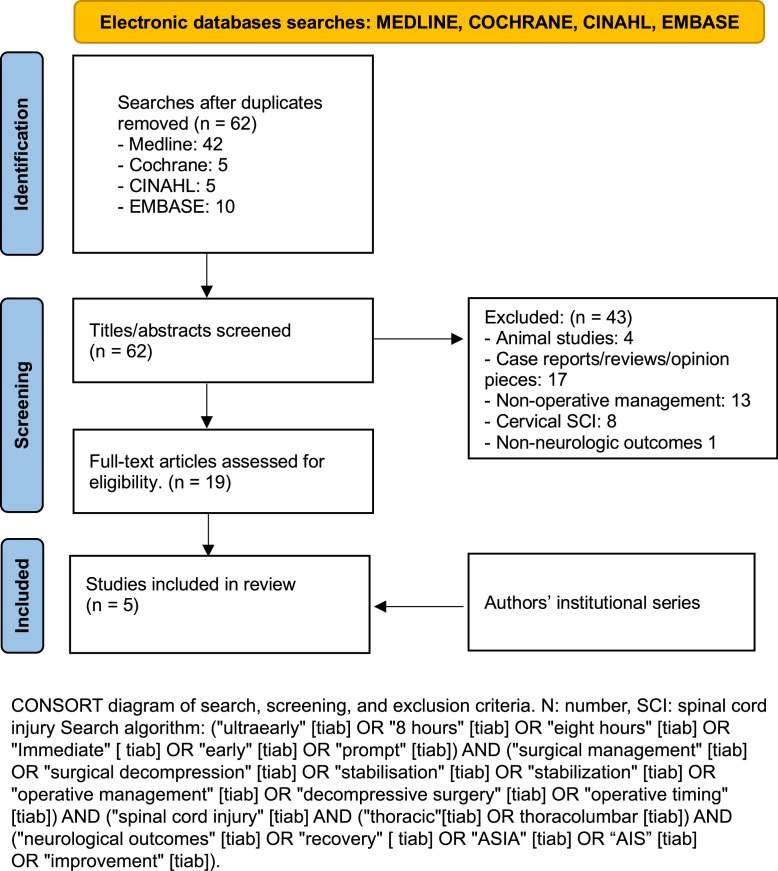
PRISMA flow chart depicting search and screening for the study.

The search algorithm resulted in 62 unique studies from 4 databases. These isolated studies were reviewed by two independent reviewers for title and abstract. Forty-three studies were excluded based on the pre-defined exclusion criteria (4 animal studies, 17 case reports/reviews/opinions, 13 nonoperative management, 8 cervical spinal cord injury, and 1 with non-neurologic outcomes). The remaining 19 studies underwent full-text screening for final inclusion. The institutional case series was added to the final study results. The PRISMA flow diagram in Fig. 1 summarizes the search strategies well as the search algorithm.

The selected patients were divided into 2 groups Ultra Early (operated within 8 hours of injury) and Control population (operated after 8 hours of injury). Demographic information was collected regarding the patient age and sex. AIS information on presentation and after intervention was also collected. AIS improvement was assigned a numerical scale, where an improvement to the next AIS grade was given the numerical value of 1. Overall, AIS improvement was treated as a mean continuous variable, although the AIS is an ordinal scale. Treating it as a continuous variable offers a concise interpretation and uses analysis techniques that are more intuitive and familiar. However, it requires the assumption that the numerical distance between the categories is equal [8], which is not unreasonable for the AIS, which is a graduated scale. Cochrane Handbook states this type of analysis is an option for ordinal data [9] and it has also been suggested for other types of analyses [10].

Both ROBINS-I [11] and RoB 2 [12] tools were used for nonrandomized and randomized studies respectively to assess the risk of bias by 2 independent reviewers. Robvis tool [13] was used to graphically visualize the risk of bias for the included studies. The authors evaluated the quality of the evidence-based on the evidence profile using the GRADE (Grading of Recommendations Assessment, Development, and Evaluation) system using 2 independent reviewers [14]. Publication bias of the included studies was assessed using Egger's regression test for funnel plot asymmetry.

### Statistical analysis

The study used RStudio Desktop version 2021.09.1 for Mac to perform an arm-based meta-analysis on all the studies to evaluate the mean difference in AIS improvement between the 2 groups. Following the arm-based analysis, the authors performed a sensitivity analysis in the form of contrast-based meta-analysis using Standardized mean difference (SMD) after excluding Payer as the study did not qualify for the contrast-based analysis. The R package *pcnetmeta* [15] was used for arm-based meta-analysis while the package *metafor* was used for contrast-based meta-analysis. A measure of heterogeneity was performed using I^2^, with a threshold for I^2^ >50% considered as significantly high heterogeneity. The statistical outcomes were reported as effect size, treatment effect, and effect difference, all with 95% confidence intervals (CI). A p-value of <.05 was considered statistically significant.

## Results

### Demographics and intervention

One hundred thirty-three patients were included in 4 studies. About 74.4% of patients were male and 25.6% were female. Seventy-six (57.1%), underwent decompression ≤ 8 hours, while 57 (42.9%) underwent decompression after 8 hours from injury.

Cengiz et al. performed a prospective randomized study on 27 patients with thoracolumbar spinal cord injury that were randomized based on presentation to the hospital on Friday or Monday, resulting in an early intervention group ≤ 8 hours and a late group operated between 3 and 15 days [16]. The AIS grade in their study was converted to numerical values of 1 to 5 corresponding to A to E, which was then used both as a categorical variable and a continuous variable by the study calculating mean AIS score upon admission and discharge.

Payer [17] present a prospective observational study for patients with thoracolumbar burst fractures, 14 of whom presented with neurological deficits and underwent posterior decompression within 8 hours of injury. The study used a categorical change in AIS scores for the patients that underwent decompression. The study did not include a control arm.

Ramirez et al. [18] is a retrospective study of 28 patients with unstable thoraco-lumbar injury with neurological symptoms, of which 11 patients underwent surgery within 8 hours and the rest were operated after 8 hours. AIS improvement was converted to numerical value and the study focused on mean AIS improvement between the ultra-early and control groups. In retrospect, treating the AIS as a continuous variable allowed us to include this particular study that also treated AIS as numerical.

Wutte et al. [19] performed a retrospective analysis of 12-year data from patients with thoraco-lumbar injury operated at a level 1 trauma center. The patients were operated as soon as possible, with 35 in the ultra-early group compared to 23 patients operated after 8 hours. The study recorded the initial AIS grade on presentation as well as the AIS score 1 year after the injury. AIS improvement was converted into a numerical scale, but the study did not calculate the mean improvement.

Carr et al. refers to the institutional case series of the author's home institution, where 6 patients with thoracolumbar spinal cord injury were analyzed, 4 of which underwent surgical decompression within 8 hours. The AIS scores were recorded as categorical improvement and as a numerical variable similar to Ramirez et al. Both Payer and Ramirez et al. gave steroids according to NASCIS II protocol [20]. Cengiz used methylprednisolone according to an established protocol during the time of the study publication [21]. Wutte et al. did not specify the use of steroids in their patients. Carr et al. used dexamethasone for the patients per the clinical judgment of the primary surgeon.

### AIS improvement

**Fig. 2:**
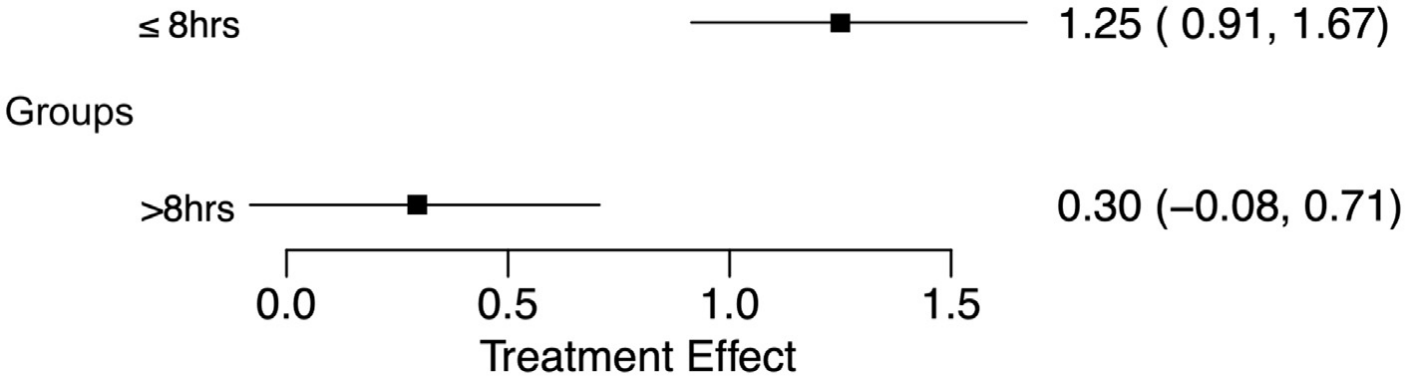
Treatment effect graph showing a statistically significant effect size for the ultra-early group in mean AIS improvement after intervention. The control group does not have a statistically significant effect size.

**Fig. 3:**
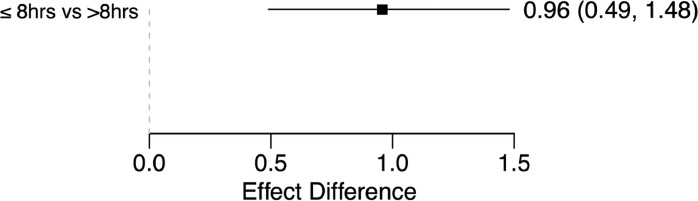
Effect difference graph showing a statistically significant difference between ultra-early and control groups.

**Fig. 4:**
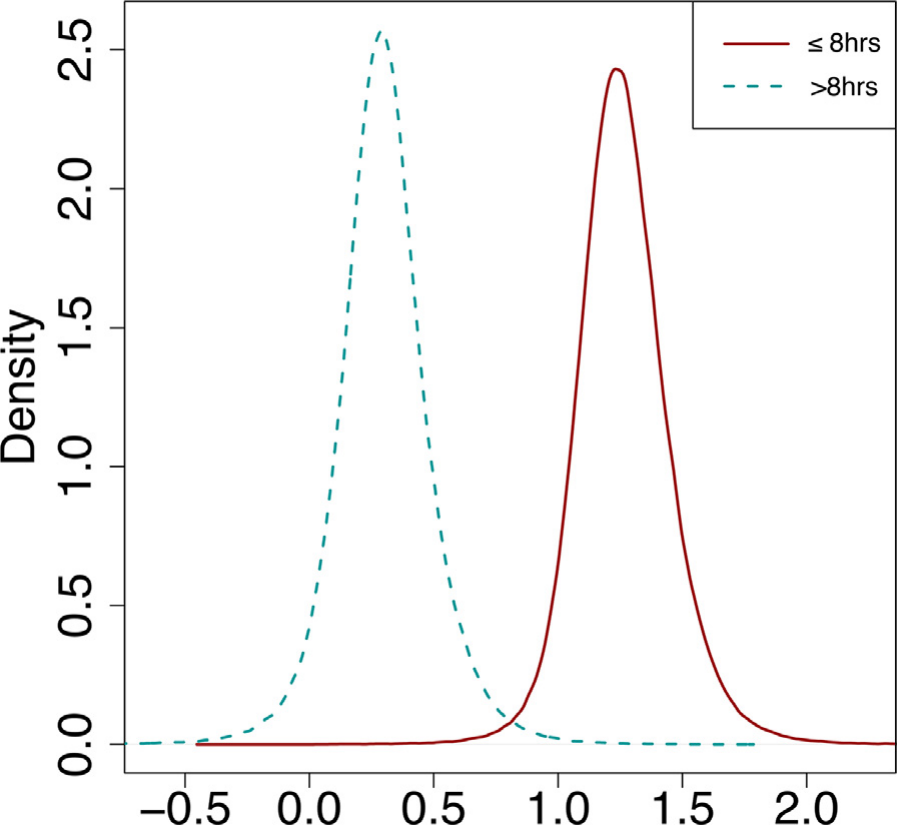
Treatment effect graph to visualizing minimal overlap between the two groups.

**Fig. 5:**
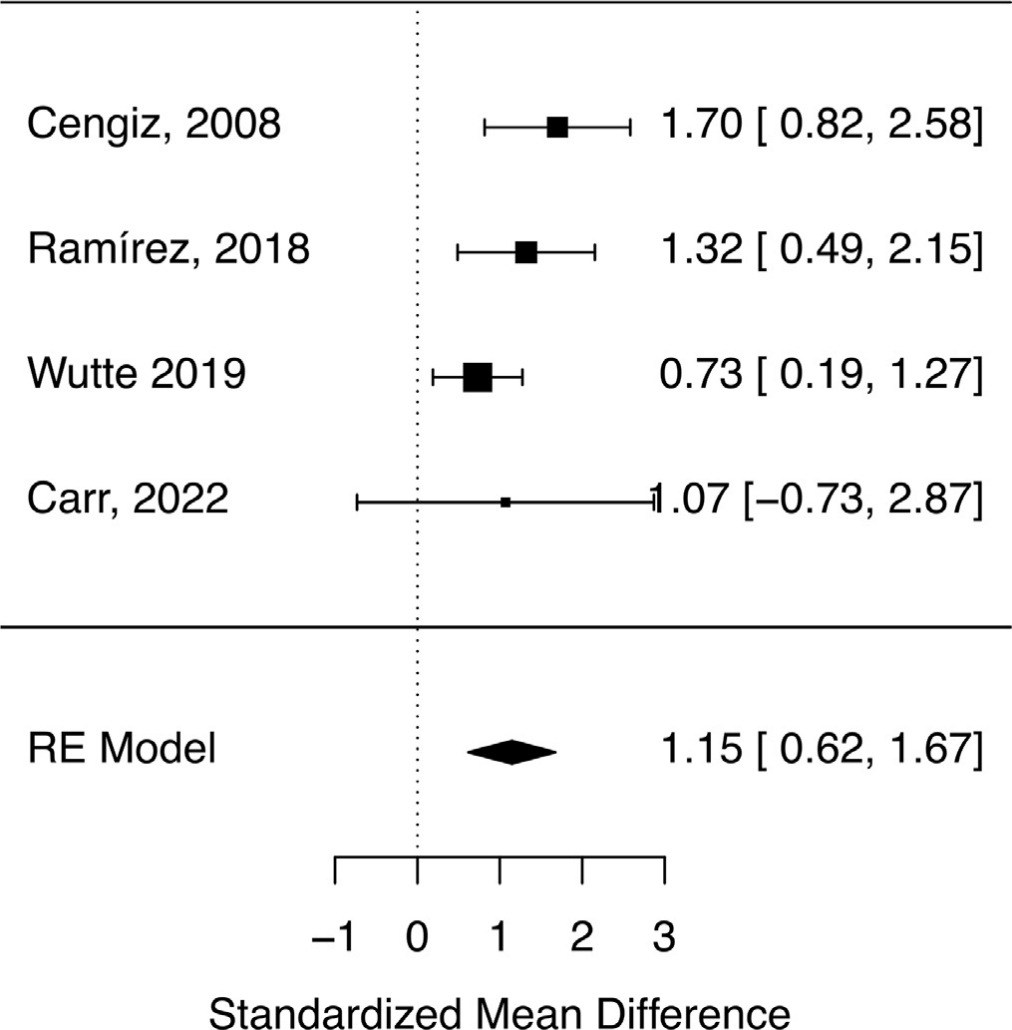
Forest plot for contrast-based meta-analysis showing statistically significant mean AIS improvement in the ultra-early group.

In the single-arm meta-analysis, there was a statistically significant treatment effect corresponding to AIS improvement in the ultra-early arm (Effect size 1.25 [0.91–1.67]), while >8-hour arm did not show significance (Effect size 0.30 [-0.08-0.71]) as depicted in Fig. 2. Moreover, the effect difference between the 2 groups (Fig. 3) was statistically significant (Effect difference 0.96 [0.49–1.48]). The significant difference between the groups is better depicted in the treatment effect density plot (Fig. 4). The contrast-based sensitivity meta-analysis using standardized mean difference (SMD) showed a significant difference in mean AIS improvement in the ultra-early group (Effect size 1.15 [0.62–1.67], p<.0001) (Fig. 5). An I^2^ value of 33.38% was obtained, indicating relatively homogeneity of the studies with consistently improved AIS outcomes in ultra-early groups.

### Risk of bias assessment

**Fig. 6:**
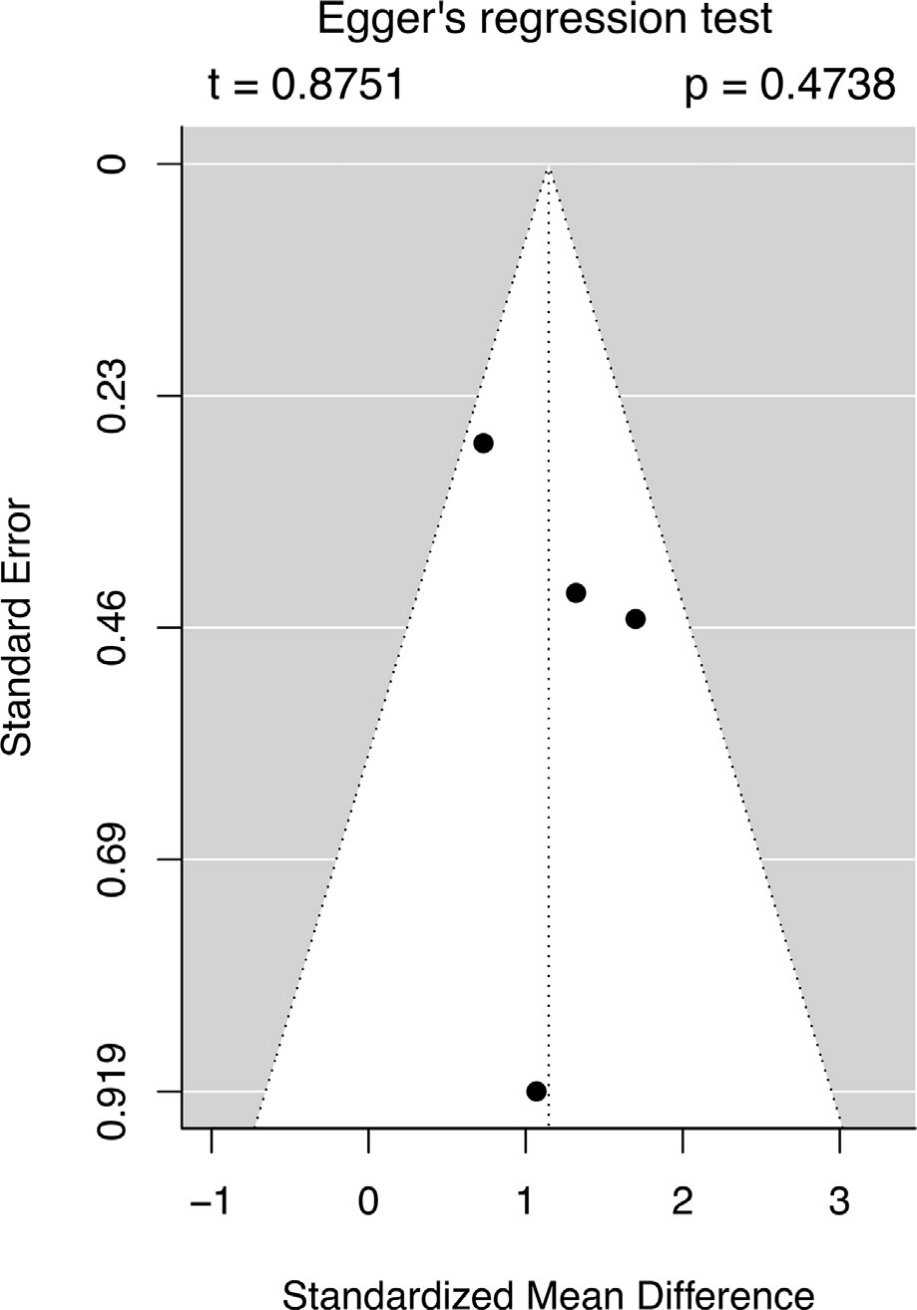
Funnel plot for contrast-based meta-analysis showing no statistically significant asymmetry, indicating no significant publication bias.

**Fig. 7:**
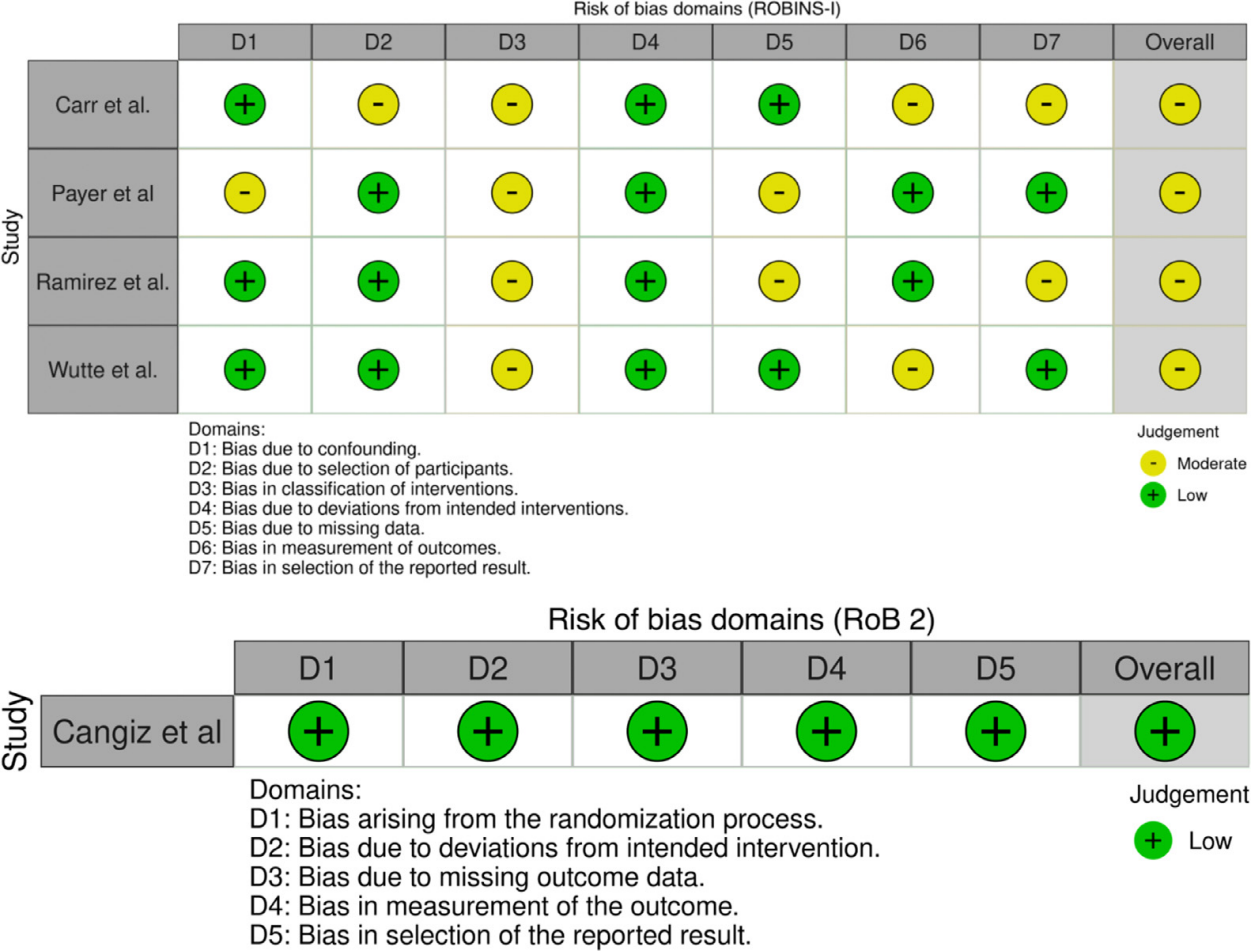
Graphical representation of risk of bias analysis using the robvis tool for studies analyzed by ROBINS-I and RoB 2 tools.

A Risk of bias assessment was performed using available tools for all the studies. Cengiz et al. showed an overall low risk of bias after evaluation with RoB 2 tool. Being nonrandomized studies, Payer et al., Ramirez et al., and Wutte et al. were evaluated by ROBINS-I tool, which concluded them as having overall moderate risk of bias. Carr et al. were also evaluated by ROBINS-I and found to have a moderate risk of bias. Egger's regression test for funnel plot asymmetry **(**Fig. 6) did not show any significant risk of bias in the contrast-based studies, with t=0.8751 and p=.4738. Fig. 7 contains graphical representation created by using robvis tool for the studies. Moreover, an analysis of the evidence profile for this study using the GRADE system is presented in Table 2.

## Discussion

### Determination of ultra-early timing

Current recommendations emphasize early (<24 hours) surgical decompression of all spinal cord injuries (SCI), irrespective of injury level or severity [22]. Urgent decompression of SCI is intended to restore blood flow and spinal cord perfusion pressure, thereby halting the cascade of secondary injury. Although the 24-hour time frame from the time of injury was chosen arbitrarily as the “line in the sand” defining early surgery, there is a case for determining whether even earlier surgical decompression could offer further benefits [23], [24], [25].

Low-level evidence indicates that “ultra-early” decompression in SCI predicts favorable neurological outcomes. Nonetheless, the relevant literature is rife with controversies, as follows: (1) The time threshold for “ultra-early” is inconsistent, spanning between 4 and 12 hours, (2) the prognostic significance of injury location is still uncertain, and (3) The effect on the injury severity at baseline is a potential confounding variable under investigation. In the clinical and academic experience of the authors, an 8-hour cut-off is a realistic and clinically feasible time window for an institution to implement if ultra-early decompression were to be made a standard of care.

### Interpretation of AIS differences

The American spinal injury association impairment scale (AIS) grade is the most frequently used scoring system in assessing the level of baseline injury and response to decompression as even a one-point change in the AIS score indicates a clinically significant improvement [26]. Thus far, the conclusions on the effect of initial injury severity on response to ultra-early decompressive surgery following SCI are conflicting. Some authors concluded that ultra-early decompression is more advantageous than early or late (>24 hours) decompression for AIS type A injuries – total motor and sensory loss – while reporting no such benefits in other AIS severities (B-D) [22]. According to one theory, the spinal cord of AIS A patients is subjected to a considerable amount of pressure, and early decompression may prevent additional damage. On the other hand, some studies indicated that surgery within 12 hours led to a statistically significant improvement in neurological recovery for AIS B-D, but not for AIS A. These opposing conclusions indicate the presence of unaccounted-for, underlying, confounding variables, warranting further investigation [16].

This meta-analysis comprised 5 studies (133 patients) that reported neurological outcomes after ultra-early (8 hours) decompressive surgery for thoracolumbar SCI (tSCI); 1 randomized controlled trial (RCT) [16], 1 prospective cohort [18], and 3 case series, including the authors’ yet-to-be-published institutional data [17]. Every study concluded that the ultra-early (< 8 hours) group experienced superior neurological recovery. The pooled estimate, which included both single-arm and contrast-based analysis, also demonstrated that ultra-early decompressive surgery is associated with improved neurological recovery. Only one paper, by Ramirez et al. [18] adjusted observed neurological outcomes to the severity of injury at baseline, and thus a meta-regression analysis could not be completed, leaving this as a significant confounding variable. As to surgical technique, the majority of the patients underwent decompression/fixation, predominantly through posterior or combined approaches. All studies, including our institutional series, documented the use of steroids perioperatively.

The RCT by Cengiz et al. found that late surgery was associated with a significant increase in the rate of complications, but there was no difference in mortality. Hospital stays were also shorter for patients who underwent surgery within 8 hours of injury in that same study. The study by Payer et al., which focused primarily on differences in outcomes in relation to operative technique, reported a complication rate of 21.4% [16]. Ramirez et al. did not report rates of complications or lengths of hospital stays, and thus a pooled estimate for these parameters could not be calculated.

It is important to acknowledge that even if we were to summon compelling evidence in support of ultra-early decompressive surgery for tSCI or even other levels of SCI, wide-scale implementation would not be an easy undertaking and we would continue to encounter an array of challenges. According to a survey of Canadian surgeons, only 34% of patients with tSCI were operated on within 12 hours of injury, with delays in transfer from the site of injury and a lack of operating rooms and personnel cited as the primary culprits [27]. In less developed regions, financial constraints, equipment accessibility, and the absence of specialized trauma facilities with multidisciplinary teams are aggravating factors. In addition, polytrauma patients with life-threatening injuries or hemodynamic instability may not be eligible. Nonetheless, if convincing evidence accumulates to support the efficacy of ultra-early decompression in promoting neurological recovery after SCI, then our practices will have to adapt to meet the demand while undeniably battling against multiple foreseen obstacles, analogous to the journey of thrombectomy interventions for ischemic stroke patients.

### Limitations

In a similar vein, several limitations are not to be overlooked in this meta-analysis. First, most of the included studies were retrospective observational studies, which is expected given the ethical constraints surrounding randomization and outcome assessment blinding. Second, the collected literature was not limited to tSCI, and all but one study characterized thoracolumbar injuries as a single group, a degree of heterogeneity that is inescapable given the anatomical and mechanical overlap between the thoracic and thoracolumbar spine. Moreover, there is a possibility of a higher percentage of spinal shock in the ultra-early group, which would make their results appear more favorable compared to the late group with improved/resolved spinal shock, which can create a ceiling effect. Given the scarcity of literature and primary studies, this meta-analysis does not have enough data from the primary sources to be able to break down differences/improvements by individual AIS grading. The overall sample size of this meta-analysis is small, with only 133 patients included, which also limits its power and generalizability. In view of the above confounding factors, future larger controlled studies are required to investigate the efficacy of ultra-early decompression on neurological recovery in cases of tSCI.

## Conclusions

The scarcity of research on ultra-early decompression for thoracolumbar spinal cord injury makes it crucial to consolidate and analyze data from existing studies. This meta-analysis shows that patients who undergo decompression within 8 hours of spinal cord injury have significantly improved mean AIS scores. These results underscore the potential of ultra-early decompression to improve patient outcomes and thus should not be overlooked.

## Declaration of Competing Interest

One or more of the authors declare financial or professional relationships on ICMJE-NASSJ disclosure forms.
